# The Biting Colon: An Unfortunate Nightmare for a Healthy but Anxious Patient

**DOI:** 10.7759/cureus.61023

**Published:** 2024-05-24

**Authors:** Jackson N Hoekstra, George Trad, Luke Johnson, Tarek Ammar, John K Ryan

**Affiliations:** 1 Internal Medicine, MountainView Hospital, Las Vegas, USA; 2 Gastroenterology, Southern Hills Hospital & Medical Center, Las Vegas, USA

**Keywords:** dental bridge, internal medicine, colonoscopy, foreign body, gastroenterolgy

## Abstract

A 48-year-old female with no significant past medical history presented to the emergency department with an uncommon scenario after accidentally ingesting a three-unit dental bridge, leading to its impaction within the lower gastrointestinal tract. Despite initial conservative management with laxatives aimed at facilitating spontaneous passage, the foreign body remained lodged in the colon. Subsequently, the patient underwent endoscopic intervention via colonoscopy, during which the dental bridge was successfully extracted. This case highlights the complexity of managing foreign body ingestions, particularly when impaction occurs in uncommon locations, such as the colon. We emphasize the importance of individualized care strategies and recognize the potential of endoscopic procedures in resolving clinical scenarios involving foreign body ingestions.

## Introduction

Foreign body ingestion is a rare occurrence in healthy adults and occurs more frequently in older adults, individuals with underlying psychiatric disease, those under the influence of alcohol, and prison inmates for the purpose of drug trafficking [1]. While foreign body ingestion is often accidental and can happen to anyone, heightened anxiety levels may contribute to altered swallowing patterns, increased muscle tension, and overall nervousness, which may increase risk of accidental ingestion and influence management decisions. Typically, 80-90% of ingested foreign bodies pass spontaneously through the gastrointestinal tract, while the remaining 10-20% may require endoscopic intervention [2-4]. The esophagus is the most commonly affected site, posing the highest risk of complications, whereas foreign body impaction in the colon is rare and carries a lower risk of complications [5,6]. Nevertheless, if a foreign body remains impacted for several days despite conservative management, invasive intervention becomes necessary to remove the object. In this report, we describe a case of accidental ingestion of a dental bridge by an anxious patient leading to impaction in the colon and subsequent endoscopic removal.

## Case presentation

**Figure 1:**
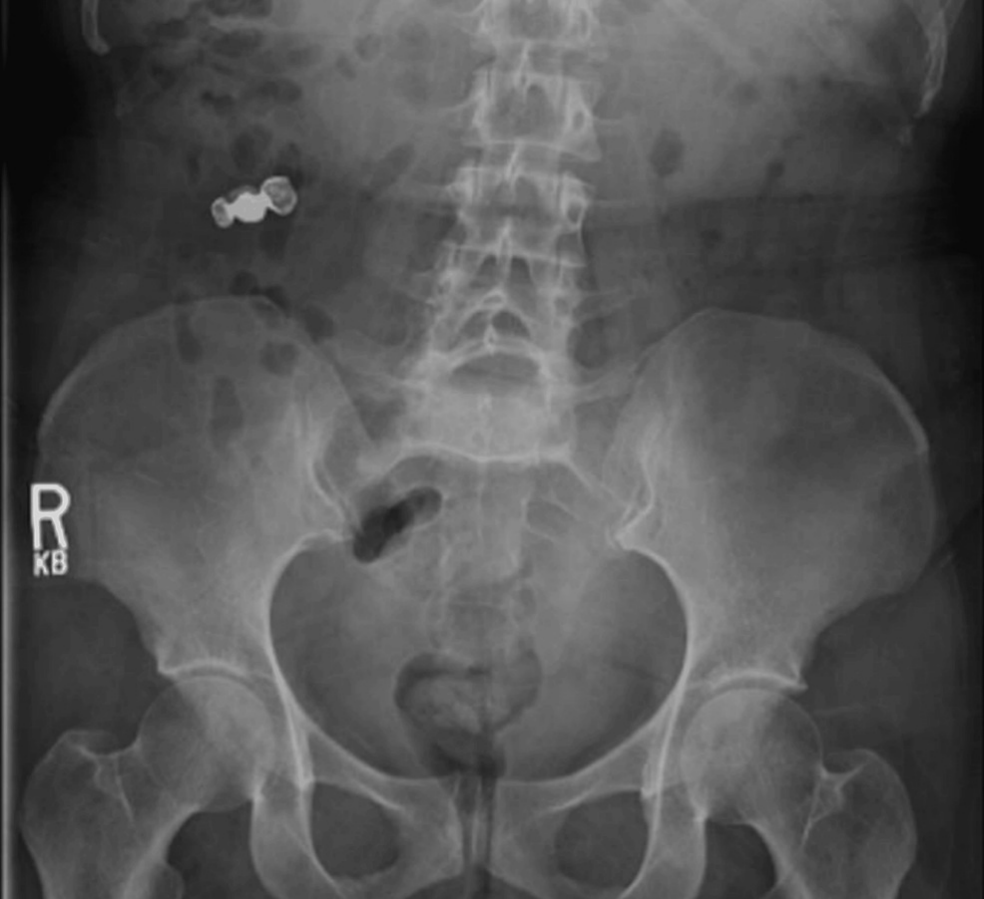
Kidney, ureter, and bladder (KUB) with a foreign body in the right lower quadrant of the abdomen

A 48-year-old female with no significant past medical history presented to the emergency department with an uncommon scenario after swallowing her three-unit dental bridge that had fallen into the back of her mouth while sleeping. She denied throat or abdominal pain and was asymptomatic. On initial presentation, vital signs were as follows: heart rate, 77 beats/min; blood pressure, 178/105 mm Hg; respiratory rate, 18 breaths/min; and oxygen saturation, 100% on room air. The physical exam was normal except for the patient appearing anxious about what happened. Initial laboratory studies were within normal limits. Chest X-ray (CXR) did not reveal a radiopaque foreign body and was otherwise normal. Kidney, ureter, and bladder (KUB) X-ray demonstrated a 2.7 x 1.3 cm right lower-quadrant metallic density, likely in the lower gastrointestinal tract (Figure 1).

Given that the dental bridge was currently in the colon, having passed the ileocecal valve, there was no plan for endoscopic intervention. The initial treatment plan was conservative, and the patient was prescribed a bowel regimen, including polyethylene glycol. Despite regular bowel movements with bowel preparation, daily KUB X-rays continued to show the foreign body in an unchanged position for three consecutive days.

**Figure 2:**
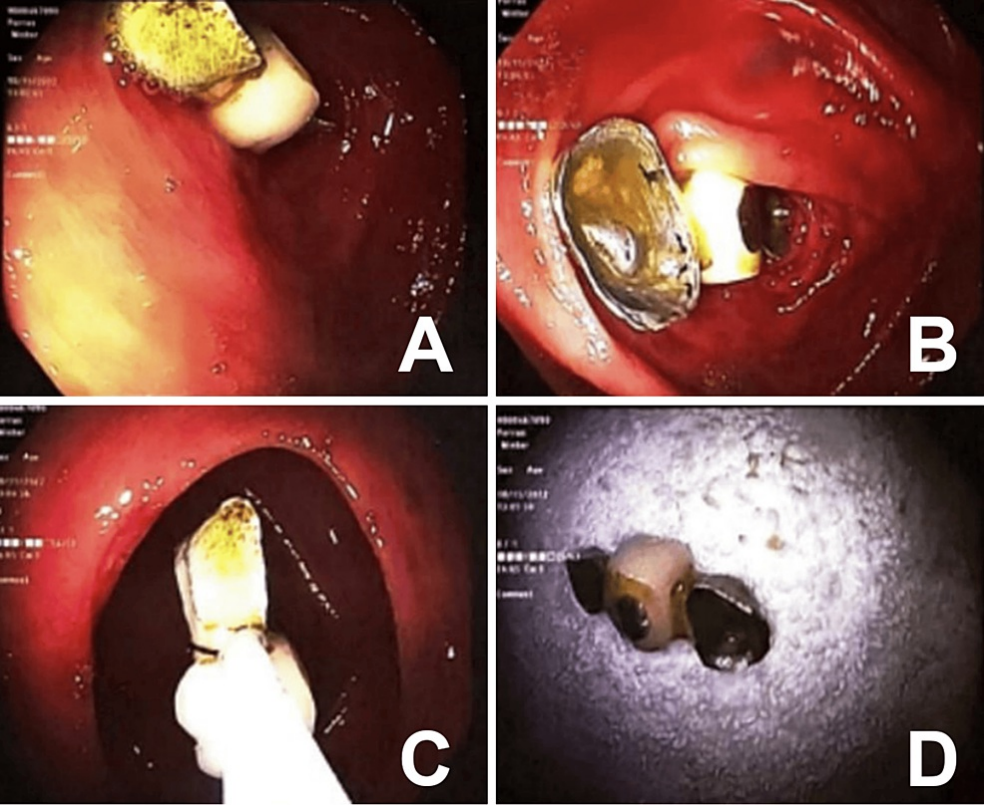
A/B: Dental bridge located at appendiceal orifice. C: Dental bridge being removed via snare through transverse colon. D: Extracted dental bridge.

The patient was highly agitated and anxious, having needed to take alprazolam frequently throughout her hospitalization. She was extremely worried that the foreign object would perforate through her colon, prompting an endoscopic extraction of the bridge unit using a colonoscope prior to being discharged. The foreign body was visualized in the vicinity of the appendiceal orifice, and the removal of the dental bridge was accomplished with a snare (Figure 2). The patient was discharged with the tooth bridge and advised to seek further follow-up with her dentist. This case highlights the need for individualized approaches in managing rare instances of foreign body ingestion with impaction in the lower gastrointestinal tract. 

## Discussion

Foreign body ingestion has a diverse clinical presentation and management plan based on the patient's history, location in the gastrointestinal tract, and the properties of the object. Foreign body ingestion is a common occurrence among children, the elderly, and mentally impaired individuals [7]. Most ingestions in children involve items like coins, toys, and batteries, while most adult ingestions are related to eating and leading to impaction [8,9]. In addition, some individuals intentionally swallow foreign bodies, mainly young males with psychiatric illnesses or involving drug abuse issues [1].

Our patient was overall asymptomatic following foreign body ingestion; however, patients may present with symptoms, such as dysphagia, odynophagia, abdominal pain, nausea, vomiting, or gastrointestinal bleeding. Presentation is dependent on the object and site of impaction [10]. Biplane radiographs are effective for detecting many foreign bodies, especially those with radiopaque metallic components, such as a metal dental bridge as in our patient [11,12]. Plain radiographs are necessary if the ingested object is not known.

Most foreign objects in the gastrointestinal tract pass within two to 12 days, with some taking up to four weeks. In our case, the object did not pass fully through the gastrointestinal tract spontaneously within four days despite an initial conservative treatment plan with laxatives. The foreign body was initially seen on the KUB in the right lower quadrant of the abdomen. In most cases with foreign body ingestion, the initial presentation commonly occurs in the stomach (58.1%) and the small intestine (32.7%) [13]. Impaction typically happens at anatomical or physiological narrowing points, like the esophageal sphincters, pylorus, duodenum, ileocecal valve, and anus [11,14,15]. The esophagus is the most common site for impaction, requiring prompt removal within 24 hours to avoid perforation [5,12]. The dental bridge in this case was visualized near the appendiceal orifice and not directly at a site commonly known for impaction.

Foreign bodies can lead to significant morbidity and mortality associated with impaction [16]. Foreign body impaction in the gastrointestinal tract poses risks, such as perforation, infection, severe bleeding, obstruction, and fistulas [11,14,17]. The overall rate of perforation owing to foreign bodies is roughly 1-7% [18]. Sharp objects carry a higher risk of complication, with the ileocecal valve and sigmoid being the most common sites of perforation [12]. Even swallowing a dental bridge can lead to intestinal perforation and may necessitate surgical resection [19].

Management of foreign body ingestion in the gastrointestinal tract depends on the object type, location, and the patient’s clinical condition. Most foreign bodies (80-90%) pass safely through the gastrointestinal tract without intervention, allowing for a conservative approach with observation, particularly if the object reaches beyond the pylorus [11,12,14,17]. Endoscopy is the preferred option for uncomplicated cases if a conservative approach fails [7]. Sharp objects should be removed endoscopically if possible or monitored closely with serial X-rays to follow the progression of the object as was the case in our patient with a three-unit dental bridge [12]. Emergent endoscopy, within two hours of arrival to the emergency department, is indicated in patients with ingestion of long sharp objects, batteries in the esophagus, and complete esophageal obstruction usually associated with food impactions [4]. Surgical intervention (<1%) is reserved for cases with complications or non-progression of the foreign body that is unable to be reached endoscopically [7]. When objects do not pass spontaneously, endoscopic or surgical removal is necessary as the longer a foreign object remains in a patient’s body, the higher the risk one has for complications, such as perforation, infection, and severe bleeding. Our patient was healthy and aware of what she swallowed and when. Although there is not a set guideline approach as to when a foreign object must be removed, a patient-centered approach that addresses the patient’s wishes as in our case is preferred.

## Conclusions

This case report highlights the importance of personalized care in managing foreign body ingestion. Our patient, a 48-year-old female, presented with a unique scenario of a dental bridge ingestion leading to colonic impaction. Initial conservative management was unsuccessful, necessitating an endoscopy intervention for removal. This case underscores the significance of recognizing diverse clinical presentations, understanding associated risks like perforation, and tailoring management strategies accordingly. The successful endoscopic retrieval of the dental bridge demonstrates the efficacy of endoscopy in resolving foreign body impaction in the lower gastrointestinal tract. The case illustrates the need for flexibility in clinical management plans to accommodate individual patient needs and responses to treatment.
